# Referral bias for specific learning disorders? The wide‐ranging challenges for the youngest in class – Commentary on Arrhenius et al. (2021)

**DOI:** 10.1111/jcv2.12013

**Published:** 2021-04-28

**Authors:** Jonna Kuntsi

**Affiliations:** ^1^ Social, Genetic and Developmental Psychiatry Centre, Institute of Psychiatry, Psychology and Neuroscience King's College London London UK

**Keywords:** specific learning disorders, relative age effect, young relative age, ADHD

The relative age effect captures the observation that children who are younger than most of their peers in the same class are at a disadvantage in relation to a range of outcomes. Arrhenius et al. (2021) point out that this phenomenon was described already in 1990; yet, one could argue it has not received the full attention among educational experts and the wider community that it surely deserves. The number of studies reporting relative age effects, for outcomes ranging from academic and sport achievement to the diagnosis of attention‐deficit/hyperactivity disorder (ADHD), continues to grow.

What is particularly notable about the research on the relative age effect is how consistent the findings are, overall, across countries with very different school systems. Having myself attended school in Finland where you start school rather late—in the year that you turn seven years old (as Arrhenius et al. also describe)—it was quite a shock to realise, as a parent living in the United Kingdom, that my late summer‐born daughter was to start school when she had only just turned 4 years old. Yet, whether children have started school at the age of 4 or 7, studies report relative age effects. The evidence also suggests that the relative age effect is not due to season of birth. For example, for ADHD diagnoses, cross‐country and cross‐state comparisons indicate that the differences in outcomes relate to school year cut‐offs rather than birth month per se (Elder, 2010; Karlstad et al., 2017; Layton et al., 2018).

Few methodologically strong studies have examined the relative age effect in relation to specific learning disorders—difficulties with reading, spelling or arithmetic skills that are not due to low general cognitive ability. I was, therefore, pleased to find out that Arrhenius et al. (2021) now address this gap, using nationwide data from the Finnish registers. Nationwide registers are an ideal source for examining such effects across very large, representative populations. The relative age effect they report for a diagnosis of a specific learning disorder before age 10 is notable, with an incidence rate ratio of 1.77 for the comparison between December‐born (youngest) and January‐born (oldest) children. The pattern of results was robust, with a trend of cumulative incidences, peaking towards the end of the calendar year. The register‐based nationwide dataset is also ideal for an analysis of possible sex effects: the lack of such an effect in the analyses by Arrhenius et al. is therefore convincing and suggests that the underlying reason for the observed relative age effects applies equally to both boys and girls.

The relative age effect previously reported on ADHD is a solid observation, as shown by a recent systematic review and meta‐analysis of 20 large cohort and register studies, across many countries and continents (Holland & Sayal, 2018). As an example, in the Swedish register data, the prevalence of ADHD diagnosis was 2.8% among the youngest in class (November–December births) and 1.7% among the oldest in class (January–February births) (Halldner et al., 2014). Given such observations and the frequent co‐occurrence of ADHD and specific learning disorders, it is important that Arrhenius et al. (2021) were able to show that the association of young relative age with specific learning disorders was not due to comorbid ADHD.

How do we explain the relative age effects? In some of the ADHD studies the relative age effect has emerged stronger for teacher‐reported ADHD symptoms, compared to parent reports, raising the possibility that teacher perceptions of immaturity relative to peers may contribute to over‐diagnosis of ADHD among the youngest in class (Elder, 2010; Holland & Sayal, 2018). As ADHD reflects the extreme of a continuous trait(s) and as ADHD diagnosis relies on a relative comparison to other children of the same age, a child with moderate ADHD symptoms but who is young‐in‐class could more easily appear to cross the threshold to diagnosis if inaccurately compared to relatively older peers. (It is important to emphasise here the issue that the relative age effect on ADHD raises is not about the overall validity of the ADHD diagnostic construct, which is shown by a wide range of consistent evidence of, for example, aetiological and neurobiological risk factors, a characteristic pattern of developmental changes and outcomes, prediction of treatment response, and associated significant clinical and psychosocial impairments [Asherson et al., 2010; Faraone et al., 2015]. Instead, the relative age effect raises the issue of how to improve accuracy of diagnosis in borderline cases, and the importance of careful age‐match comparisons.) The study by Arrhenius et al. (2021) now shows that relative age effects emerge even for the diagnoses of specific learning disorders that are based on standardised, age‐adjusted psychological tests. The overall pattern of the findings leads the authors to suggest, convincingly in my view, that the most likely explanation for the relative age effect on the diagnosis of specific learning disorders is referral bias.

We must not underestimate the importance of this possibility that children may be more likely to be referred for a specific learning disorder assessment if they are among the youngest in the class, and less likely to be referred for such an assessment if they are among the oldest in the class. No doubt we are all in agreement that access to a specialist educational assessment and subsequent intervention—and the related phenomena such as those relating to potential negative consequences of ‘labelling’—should not relate to the child's month of birth.

How could we avoid such referral bias? Arrhenius et al. (2021) suggest, firstly, that education professionals should ‘keep the educational disadvantages of the relatively young in mind when evaluating learning capabilities’. This is important, but may not always be easy to achieve in practice. Their second suggestion is that ‘greater flexibility in school admissions for less mature children might be warranted’. The authors refer to the Danish school system that has shown benefits for some relatively younger children—girls in particular—of being held back by a year. Interestingly, in the studies on the relative age effect on ADHD, significant effects emerged from the Nordic register‐based studies from Sweden, Finland, Norway and Iceland, but the Danish dataset was an exception (Pottegård et al., 2014), in showing almost no relative age effect on medication use for ADHD. The most likely explanation for the lack of a relative age effect in Denmark is indeed the more flexible approach to school starting age. Arrhenius et al. also rightly point out potential challenges in such an approach, however. More research on the benefits and potential disadvantages of a more flexible approach to school starting age, including detailed cross‐country comparisons, is undoubtedly needed.

Could we also consider the feasibility of wider, systematic initial screening at certain precise ages? Could standardised measures of reading, spelling and arithmetic skills be used in schools systematically to objectively select those above a specific screening cut‐off who would be referred for a specialist assessment? In theory at least, such a practice could help avoid teachers inadvertently being more likely to select the most developmentally immature—the youngest in class—for the specialist assessments. An educational debate—across country borders—is now needed to review approaches that could best ensure that children's future outcomes are fully independent of their relative age at the start of school.

## CONFLICTS OF INTEREST

The author is on the Editorial Advisory Board of JCPP *Advances*. [Corrections made on 22 June 2022, after first online publication: This Conflicts of Interest statement has been corrected in this version.]
